# Gelsemium Sempervirens

**Published:** 1883-08

**Authors:** 


### BOOK REVIEWS.

Gelsemium Sempervirens. A Monograph. By the Hughes Medical Club of Massachusetts. Boston: Otis Clapp & Son. 1883.

Gelsemium is a very important drug; it occupies no mean position in our therapeutics. But one who knew not its clinical value would fail to give to Gels. its true worth if he judged it simply by this manual. By rejecting clinical symptoms (often confirmatory and complementary to the pathogenetic) and those from the potencies, the Hughes Club has much diminished the value of their monograph. Indeed, this monograph is merely a correction of previous knowledge, not *adding* to but subtracting from it! Would it not have been more useful had this Society made new provings?

The Austrian provers, when studying Natrum muriaticum, re-proved it, which was much more useful than criticising, or shall we call it *correcting*, others.

The book is well arranged, symptoms well stated, and is very neatly gotten up.

